# French Bulldogs differ to other dogs in the UK in propensity for many common disorders: a VetCompass study

**DOI:** 10.1186/s40575-021-00112-3

**Published:** 2021-12-16

**Authors:** Dan G. O’Neill, Rowena M.A. Packer, Peter Francis, David B. Church, Dave C. Brodbelt, Camilla Pegram

**Affiliations:** 1 Pathobiology and Population Sciences, The Royal Veterinary College, Hawkshead Lane, North Mymms, AL9 7TA Hatfield, Herts UK; 2 Clinical Science and Services, The Royal Veterinary College, Hawkshead Lane, North Mymms, AL9 7TA Hatfield, Herts UK

**Keywords:** VetCompass, Electronic patient record, Primary-care, Purebred, Predisposition, Brachycephalic, Flat faced

## Abstract

**Background:**

The French Bulldog is a highly popular dog breed but is linked with many serious health issues. A holistic view of breed health in French Bulldogs would assist efforts to appreciate the overall health strengths and weaknesses in the French Bulldog and to take appropriate steps to mitigate these.

Based on random sampling of French Bulldogs and non-French Bulldogs under primary veterinary care during 2016 within the VetCompass Programme, a cohort study design was used to estimate the one-year (2016) period prevalence of the most commonly diagnosed disorders in each group. Risk factor analysis used multivariable logistic regression modelling methods.

**Results:**

The analysis included 2,781 French Bulldogs and 21,850 non-French Bulldogs. French Bulldogs were younger (1.51 years, IQR 0.86 – 2.77 vs. 4.48 years, IQR 1.94 – 8.14) (*p* < 0.001) and lighter (12.45 kg, IQR 11.00 – 14.03 versus 13.80 kg, IQR 8.10 – 25.12) (*p* < 0.001) than non-French Bulldogs. Of 43 common specific-level disorders across both groups, French Bulldogs had significantly increased adjusted odds of 20/43 (46.5 %) disorders and significantly reduced adjusted odds of 11/43 (25.6 %) disorders compared to non-French Bulldogs. Highly predisposed disorders in French Bulldogs included stenotic nares (OR 42.14; 95 % CI 18.50 to 95.99; *p* < 0.001), Brachycephalic Obstructive Airway Syndrome (OR 30.89; 95 % CI 20.91 to 45.64; *p* < 0.001), aural discharge (OR 14.40; 95 % CI 9.08 to 22.86; *p* < 0.001), skin fold dermatitis (OR 11.18; 95 % CI 7.19 to 17.40; *p* < 0.001) and dystocia (OR 9.13; 95 % CI 5.17 to 16.13; *p* < 0.001). At a grouped-level of diagnostic precision, French Bulldogs had increased adjusted odds of 12/32 (37.5 %) disorders and reduced adjusted odds of 6/32 (18.8 %) disorders compared to non-French Bulldogs.

**Conclusions:**

These results identified ultra-predispositions with worryingly higher odds in French Bulldogs for several disorders, suggesting that the health of French Bulldogs has diverged substantially from, and may be lower than, the health of the wider non-French Bulldog population. Many of these predispositions are closely associated with the conformational extremes that define the French Bulldog breed. Shifting the typical conformation of the French Bulldog population towards a more moderate phenotype is proposed as a logical opportunity to reduce the serious health issues endemic in the French Bulldog breed.

## Plain English Summary

The French Bulldog is currently a hugely popular dog breed in the UK. However, the breed is linked with a range of serious health issues. Using veterinary clinical data from the VetCompass Programme at the Royal Veterinary College, this study aimed to compare the frequency of common disorders in French Bulldogs against that of all remaining dogs to identify health strengths and weaknesses in French Bulldogs. This overall view of breed health can assist owners, breeders and veterinarians to take appropriate actions to improve the health of French Bulldogs.

From an overall population of 905,544 dogs, random samples of 2,781 French Bulldogs and 21,850 non-French Bulldogs were included in the analysis. French Bulldogs were younger (1.51 years versus 4.48 years) and lighter (12.45 kg versus 13.80 kg) than non-French Bulldogs. French Bulldogs had increased risk of 20/43 (46.5 %) specific disorders and decreased risk of 11/43 (25.6 %) specific disorders compared to non-French Bulldogs. The disorders with greatest relative risk in French Bulldogs compared to non-French Bulldogs were narrowed nostrils (x 42.14), Brachycephalic Obstructive Airway Syndrome (x 30.89), ear discharge (x 14.40), skin fold dermatitis (x 11.18) and difficulty giving birth [dystocia] (x 9.13). When the disorders were grouped into broad disease categories, French Bulldogs had increased risk of 12/32 (37.5 %) disorder groups and reduced risk of 6/32 (18.8 %) disorder groups compared to non-French Bulldogs.

This study suggests that the health of French Bulldogs is very different, and largely much poorer, that the health of the wider non-French Bulldog population. Many of these differences are closely associated with the extreme body shape that defines the French Bulldog breed. Shifting the body shape of French Bulldogs to become more moderate, and hence less extreme, is proposed as a logical opportunity to reduce the current serious and common health issues in the French Bulldog breed.

.

## Background

The French Bulldog has shown phenomenally rising popularity in the UK over the past decade, recording a twenty-fold increase in Kennel Club registrations from 1,521 to 2009 to 33,661 in 2019 and becoming the second most commonly registered breed in 2019 (behind the Labrador Retriever) [1]. Surprisingly, however, public demand for French Bulldogs has risen in parallel with increasing availability and public dissemination of evidence on an array of health issues affecting the breed [2–4]. Concern over the paradox between rising popularity of certain brachycephalic breeds, such as the French Bulldog, and a growing evidence base on serious health issues that can harm the welfare and quality of life of brachycephalic dogs, led to the establishment of the Brachycephalic Wording Group in 2016 as a UK national coalition of welfare-focused organisations [5]. Within efforts to encourage owners to incorporate health criteria during their decision-making on breed selection, and with an aim to reduce demand for breeds with extreme brachycephalic conformation such as the French Bulldog, the UK Brachycephalic Working Group promotes a wide public message that recommends ‘Stop and think before buying a flat-faced dog’ [5]. Although owners of brachycephalic dogs often acknowledge the existence of serious health issues in breeds such as French Bulldogs, these owners remain highly bonded to these breeds and show enduringly high tendencies to recommend and to re-purchase these breeds regardless in the future [6, 7]. In support of the aims of the UK Brachycephalic Working Group to provide a robust evidence base on the overall health of individual brachycephalic breeds, the current study was designed to provide a holistic view of breed health in French Bulldogs compared to other dogs.

*Disorder predisposition* describes increased susceptibility and can result from genetic (hereditary) or other risk factors (e.g., breed, age, environment) [8]. Conversely, *disorder protection* describes an affinity to evade a specific condition [9, 10]. Predispositions have been previously reported in French Bulldogs for 17 disorders affecting a range of body systems [11]. Given the extremely brachycephalic conformation of the French Bulldog [12], it is unsurprising that many of these reported predispositions relate to the severely flattened skull phenotype of the breed, including issues around brachycephalic obstructive airway syndrome [13], upper respiratory tract disorders [14], corneal ulceration [15], prolapse of the nictitating membrane [16] and stenotic nares [17]. French Bulldogs have also been reported with predispositions to other disorders including hemivertebrae and vertebral kyphosis [18, 19] dystocia [20], elbow dysplasia [21], patellar luxation [22], skin fold dermatitis [23], screw tail [24] and demodicosis [25]. However, although these previous reports provide some useful information, it is not easy to prioritise the welfare impacts from these predisposed disorders on French Bulldogs overall because these earlier results derived from such a diversity of studies with diverse sample sizes, source populations, comparator groups, case definitions and study designs [26]. A fuller exploration of both predispositions and protections across the full range of disorders within a single dataset would offer a truer overall picture of health of the breed. However, to date, few studies have been published that were designed specifically to identify disorder protections [27].

Support for the generation of new information on disorder predispositions and protections is currently of special interest to The Kennel Club in the UK in order to support its programme of Breed Health and Conservation Plans (BHCP) [28]. The Breed Health and Conservation Plans culminate from the combined efforts across a broad spectrum of stakeholders including academic researchers, The Kennel Club, breed clubs and breeders to develop breed-specific health plans that can support strategies to prioritise and tackle the important health issues of individual breeds. Research data on the health of each breed is identified and collated to prioritise the most significant health issues for that breed. Based on this information, conclusions are drawn and guidance is generated on how to improve breed health. Where data gaps are identified, these are prioritised for future research to fill. The current study was designed with a view to meeting the informational needs of the BHCPs for French Bulldogs.

Secondary application of first opinion veterinary clinical data as a research resource that can give useful insights into the health of companion animals is now well established [29]. A growing number of research programmes are taking this research approach in countries such as the UK [30, 31], Netherlands [32] and Australia [33]. Research using first opinion veterinary clinical data benefits from reduced selection bias compared with referral veterinary, insurance and survey data [34]. Based on the VetCompass™ database of first opinion clinical records [30], the current study aimed to evaluate the overall health of French Bulldogs in the UK by comparing the nature and counts of predispositions and protections in French Bulldogs compared to non-French Bulldogs after accounting for demographic confounding variables. Based on the published literature supporting serious health issues that suggest a negative balance of health in the breed, it was hypothesised that the count of disorder predispositions is greater than the count of disorder protections in French Bulldogs. These results could assist breeders, veterinary practitioners and owners with a robust evidence base on the relative health of the general population of French Bulldogs dogs in order to better predict, prevent and manage key health and welfare opportunities.

Methods.

The study population included all dogs under primary veterinary care at clinics participating in the VetCompass Programme during 2016. Dogs under veterinary care were defined as those with either (a) at least one electronic patient record (EPR) (VeNom diagnosis term, free-text clinical note, treatment or bodyweight) recorded during 2016 or (b) at least one EPR recorded during both 2015 and 2017. VetCompass collates de-identified EPR data from primary-care veterinary practices in the UK for epidemiological research [30]. Data fields available to VetCompass researchers include a unique animal identifier along with veterinary group, species, breed, date of birth, sex, neuter status, insurance status and bodyweight, and also clinical information from free-form text clinical notes, summary diagnosis terms [35] and treatment with relevant dates.

A cohort study design was used to estimate and compare the one-year (2016) period prevalence of the most commonly diagnosed disorders in a randomly selected sample of French Bulldogs and a randomly selected sample of all remaining dogs. Power calculations estimated that 2,184 French Bulldogs and 21,832 non-French Bulldogs were required to detect an odds ratio of ≥ 1.5 for a disorder occurring in 2 % of the non-French Bulldogs, with 80 % power and 95 % confidence and assuming a 10:1 ratio of non-French Bulldogs to French Bulldogs in the study population (Epi Info 7 CDC, 2019, O’Neill et al., 2014b). Ethics approval was obtained from the RVC Ethics and Welfare Committee (reference number SR2018-1652).

Breed information entered by the participating practices was cleaned and mapped to a VetCompass breed list derived and extended from the VeNom Coding breed list [35]. Dogs recorded as French Bulldog were categorised as French Bulldog and dogs recorded with any other breed term were categorised as non-French Bulldog. Neuter status was defined by the final available EPR neuter value and was combined with sex to generate a sex-neuter variable: female entire, female neutered, male entire and male neutered. Adult bodyweight was defined as the mean of all bodyweight (kg) values recorded for each dog after reaching 18 months old. Mean adult bodyweight was reported overall and broken down by sex for all breeds with adult bodyweight available for at least 100 dogs. Bodyweight was further categorized as “at or above the breed/sex mean”, “below the breed/sex mean” and “no recorded bodyweight”. Age (years) at the final study date (December 31, 2016) was categorised: < 1.0, 1.0 to < 2.0, 2.0 to < 4.0, 4.0 to < 6.0, 6.0 to < 8.0 and ≥ 8.0. Veterinary group attended was categorised as 1-5, based on the 5 practice groups involved in the study. Insurance status was categorised as insured or not insured as recorded by the final available EPR.

The list of unique animal identification numbers for all dogs under veterinary care in 2016 was randomly ordered and the clinical records of randomly selected subsets of French Bulldogs and non-French Bulldogs were reviewed in detail to extract the most definitive diagnoses recorded for all disorders with evidence of existence during 2016 [29]. Elective (e.g., neutering) or prophylactic (e.g., vaccination) clinical events were not included. No distinction was made between pre-existing and incident disorder presentations. Disorders described within the clinical notes using presenting sign terms (e.g., ‘vomiting’ or ‘vomiting and diarrhoea’), but without a formally recorded clinical diagnostic term, were included using the first sign listed (e.g., vomiting). The extracted diagnosis terms were mapped to a dual hierarchy of diagnostic precision for analysis: specific-level precision and grouped-level precision as previously described [29]. Briefly, specific-level precision terms described the original extracted terms at the maximal diagnostic precision recorded within the clinical notes (e.g., inflammatory bowel disease would remain as inflammatory bowel disease). Grouped-level precision terms mapped the original diagnosis terms to a general level of diagnostic precision (e.g., inflammatory bowel disease would map to gastro-intestinal).

Following data checking for internal validity and cleaning in Excel (Microsoft Office Excel 2013, Microsoft Corp.), analyses were conducted using SPSS version 24.0 (IBM Corp). The sex-neuter status, age, adult bodyweight and insurance status for French Bulldogs and non-French Bulldogs under veterinary care during 2016 were described. One-year period prevalence values were reported separately for French Bulldogs and non-French Bulldogs to describe the probability of diagnosis at least once during 2016. The final combined list of common disorders aimed to weight each breed group equally by including all disorders that featured among the 30 most common disorders in French Bulldogs and the 30 most common disorders in non-French Bulldogs. This approach generated a combined list of 43 specific-level disorders and 32 grouped-level disorders overall. Continuous variables were non-normally distributed and were summarised using median, interquartile range (IQR) and range. Mann-Whitney U test, chi-square test and Fisher’s exact test were used as appropriate for univariable statistical comparisons [36]. Multivariable binary logistic regression modelling was used to report the adjusted odd ratios (aOR) comparing French Bulldogs with non-French Bulldogs for each disorder in the combined lists of common disorders. A separate model was created for each specific-level and grouped disorder. Information theory was applied to generate a list of confounding variables that was consistently included alongside the breed variable in each model [37, 38]. Breed was an *a priori* factor of interest and the models additionally included age (years), sex-neuter status, at/above or below mean bodyweight, insurance status and veterinary group. Model fit was assessed with the Hosmer-Lemeshow Test [39]. Statistical significance was set at the 5 % level.

## Results

### Descriptive results

The study population of 905,544 dogs under veterinary care during 2016 in the UK included 16,397 (1.81 %) French Bulldogs and 889,147 (98.19 %) non-French Bulldogs. Randomly selected samples of 2,781/16,397 (16.96 %) French Bulldogs and 21,850/889,147 (2.46 %) non-French Bulldogs were included in the analysis. Data completeness were: breed 99.7 %, age 98.7 %, sex-neuter status 99.7 %, insurance status 100.0 % and bodyweight 64.5 %.

Descriptive results were reported on 2,781 French Bulldogs and 21,850 non-French Bulldogs (Table 1). The median age of French Bulldogs (1.51 years, IQR 0.86 – 2.77, range 0.10 – 13.7) was younger than for non-French Bulldogs (4.48 years, IQR 1.94 – 8.14, range 0.01 – 20.46) (*p* < 0.001). The median bodyweight of French Bulldogs (12.45 kg, IQR 11.00 – 14.03, range 3.70 – 18.40) was lighter than for non-French Bulldogs (13.80 kg, IQR 8.10 – 25.12, range 1.41 – 85.00) (*p* < 0.001).

**Table 1:** Descriptive statistics for demographic characteristics in French Bulldogs (n = 2,781) and non-French Bulldogs (n = 21,850) under primary veterinary care in the UK. The P-value represents comparison of demographic variables between French Bulldogs and non-French Bulldogs

Variable	Category	French Bulldog count (%)	Non-French Bulldog count (%)	P-value
Age (years)	< 1	818 (30.2)	2383 (11.0)	< 0.001
	1 to < 2	847 (31.3)	3142 (14.5)	
	2 to < 4	707 (26.1)	4351 (20.1)	
	4 to < 6	230 (8.5)	3412 (15.8)	
	6 to < 8	68 (2.5)	2782 (12.9)	
	≥ 8	36 (1.3)	5547 (25.7)	
Sex-neuter status	Male entire	1053 (38.0)	6305 (28.9)	< 0.001
	Male neutered	338 (12.2)	5174 (23.8)	
	Female entire	1181 (42.6)	5484 (25.2)	
	Female neutered	200 (7.2)	4822 (22.1)	
At/above or below mean bodyweight for breed and sex	At or above	547 (19.7)	6749 (30.9)	< 0.001
	Below	627 (22.6)	7957 (36.4)	
	Not recorded	1604 (57.7)	7127 (32.6)	
Insurance status	Insured	350 (12.6)	2931 (13.4)	0.236
	Not insured	2431 (87.4)	18,919 (86.6)	
Veterinary group	1	1213 (43.6)	9892 (45.3)	0.004
	2	15 (0.5)	77 (0.4)	
	3	988 (35.5)	7155 (32.7)	
	4	137 (4.9)	975 (4.5)	
	5	428 (15.4)	3751 (17.2)	

Of the French Bulldogs, 1,764/2,781 (63.4 %) were diagnosed with ≥ 1 disorder during 2016 compared with 14,442/21,850 (66.1 %) of the non-French Bulldogs. After using multivariable methods to account for effects of age, sex-neuter status, at/above or below mean bodyweight, insurance status and veterinary group, French Bulldogs had a lower adjusted odds of diagnosis with ≥ 1 disorder than non-French Bulldogs (adjusted odds ratio [aOR] 0.89; 95 % confidence interval [CI] 0.82 to 0.97; p = 0.005).

### Specific-level disorders

The combined list of the 30 most common disorders in French Bulldogs and the 30 most common disorders in non-French Bulldogs yielded a final list of 43 common specific-level disorders. At a specific-level of diagnostic precision, after accounting for confounding using multivariable methods, French Bulldogs had significantly increased adjusted odds of 20/43 (46.5 %) specific-level disorders compared to non-French Bulldogs. These predisposed disorders included: stenotic nares (aOR 42.14; 95 % CI 18.50 to 95.99; *p* < 0.001), Brachycephalic Obstructive Airway Syndrome (aOR 30.89; 95 % CI 20.91 to 45.64; *p* < 0.001), aural discharge (aOR 14.40; 95 % CI 9.08 to 22.86; *p* < 0.001), skin fold dermatitis (aOR 11.18; 95 % CI 7.19 to 17.40; *p* < 0.001) and dystocia (aOR 9.13; 95 % CI 5.17 to 16.13; *p* < 0.001). Conversely, French Bulldogs had significantly reduced adjusted odds of 11/43 (25.6 %) specific-level disorders compared to non-French Bulldogs. These protected disorders included: undesirable behaviour (aOR 0.09; 95 % CI 0.03 to 0.24; *p* < 0.001), post-operative wound (aOR 0.11; 95 % CI 0.04 to 0.30; *p* < 0.001), retained deciduous tooth (aOR 0.16; 95 % CI 0.08 to 0.31; *p* < 0.001), lameness (aOR 0.26; 95 % CI 0.15 to 0.42; *p* < 0.001) and obesity (aOR 0.30; 95 % CI 0.21 to 0.42; *p* < 0.001) (Table 2).

**Table 2:** Specific-level of diagnostic precision: multivariable logistic regression adjusted odds ratios with corresponding 95 % CIs (confidence intervals) for the most common disorders overall in French Bulldogs and non-French Bulldogs under primary veterinary care at UK practices participating in the VetCompass™ Programme from January 1st 2016 to December 31st, 2016. Model variables accounted for included age, sex-neuter status, at/above or below mean bodyweight, insurance status and veterinary group

Specific-level disorder	French Bulldog Count (%)	Non-French Bulldog Count (%)	Adjusted odds ratio	95 % CI**	P-value
Stenotic nares	51 (1.8)	7 (0.0)	42.14	18.50 to 95.99	**< 0.001**
Brachycephalic Obstructive Airway Syndrome	153 (5.5)	44 (0.2)	30.89	20.91 to 45.64	**< 0.001**
Aural discharge	74 (2.7)	36 (0.2)	14.40	9.08 to 22.86	**< 0.001**
Skin fold dermatitis	61 (2.2)	52 (0.2)	11.18	7.19 to 17.40	**< 0.001**
Dystocia	35 (1.3)	22 (0.1)	9.13	5.17 to 16.13	**< 0.001**
Allergic skin disorder	50 (1.8)	61 (0.3)	7.68	4.97 to 11.87	**< 0.001**
Food hypersensitivity	52 (1.9)	66 (0.3)	6.96	4.61 to 10.51	**< 0.001**
Musculoskeletal injury	70 (2.5)	113 (0.5)	5.45	3.89 to 7.65	**< 0.001**
Upper respiratory tract infection	48 (1.7)	81 (0.4)	4.88	3.24 to 7.35	**< 0.001**
Demodicosis	42 (1.5)	43 (0.2)	4.63	2.91 to 7.35	**< 0.001**
Corneal ulceration	45 (1.6)	163 (0.7)	4.38	2.95 to 6.50	**< 0.001**
Dermatitis	54 (1.9)	155 (0.7)	3.54	2.50 to 5.01	**< 0.001**
Allergy	88 (3.2)	341 (1.6)	2.77	2.14 to 3.59	**< 0.001**
Gastroenteritis	91 (3.3)	286 (1.3)	2.55	1.96 to 3.30	**< 0.001**
Pododermatitis	58 (2.1)	290 (1.3)	2.45	1.80 to 3.35	**< 0.001**
Patellar luxation	57 (2.0)	227 (1.0)	2.31	1.68 to 3.17	**< 0.001**
Atopic dermatitis	38 (1.4)	243 (1.1)	2.14	1.48 to 3.11	**< 0.001**
Claw injury	58 (2.1)	302 (1.4)	2.09	1.53 to 2.83	**< 0.001**
Otitis externa	262 (9.4)	1582 (7.2)	1.57	1.36 to 1.82	**< 0.001**
Heart murmur	32 (1.2)	472 (2.2)	1.38	0.92 to 2.05	0.117
Pyoderma	41 (1.5)	316 (1.4)	1.35	0.95 to 1.92	0.091
Anal sac impaction	124 (4.5)	1058 (4.8)	1.27	1.03 to 1.55	**0.022**
Cryptorchidism	35 (1.3)	123 (0.6)	1.25	0.84 to 1.84	0.273
Conjunctivitis	71 (2.6)	487 (2.2)	1.13	0.87 to 1.47	0.373
Vomiting	91 (3.3)	654 (3.0)	1.01	0.80 to 1.27	0.957
Diarrhoea	106 (3.8)	826 (3.8)	0.82	0.66 to 1.02	0.070
Wound	26 (0.9)	246 (1.1)	0.82	0.54 to 1.25	0.363
Overgrown nail(s)	105 (3.8)	1223 (5.6)	0.74	0.60 to 0.91	**0.005**
Pruritus	28 (1.0)	355 (1.6)	0.73	0.49 to 1.08	0.117
Foreign body	31 (1.1)	278 (1.3)	0.71	0.48 to 1.04	0.080
Aggression	31 (1.1)	500 (2.3)	0.64	0.44 to 0.93	**0.021**
Skin cyst	9 (0.3)	246 (1.1)	0.64	0.32 to 1.28	0.210
Coughing	12 (0.4)	214 (1.0)	0.54	0.29 to 0.98	**0.042**
Osteoarthritis	4 (0.1)	524 (2.4)	0.52	0.19 to 1.43	0.206
Flea infestation	29 (1.0)	449 (2.1)	0.46	0.31 to 0.68	**< 0.001**
Skin mass	8 (0.3)	462 (2.1)	0.38	0.19 to 0.78	**0.008**
Periodontal disease	45 (1.6)	2788 (12.8)	0.31	0.23 to 0.42	**< 0.001**
Obesity	36 (1.3)	1568 (7.2)	0.30	0.21 to 0.42	**< 0.001**
Lameness	16 (0.6)	582 (2.7)	0.26	0.15 to 0.42	**< 0.001**
Lipoma	1 (0.0)	320 (1.5)	0.24	0.03 to 1.72	0.154
Retained deciduous tooth	9 (0.3)	223 (1.0)	0.16	0.08 to 0.31	**< 0.001**
Post-operative wound	4 (0.1)	263 (1.2)	0.11	0.04 to 0.30	**< 0.001**
Undesirable behaviour	4 (0.1)	329 (1.5)	0.09	0.03 to 0.24	**< 0.001**

### Grouped-level disorders

At a grouped-level of diagnostic precision, after accounting for confounding using multivariable methods, French Bulldogs had significantly increased adjusted odds of 12/32 (37.5 %) grouped-level disorders compared to non-French Bulldogs. These included: upper respiratory tract disorder (aOR 3.80; 95 % CI 3.25 to 4.45; p < 0.001), spinal cord disorder (aOR 3.00; 95 % CI 1.94 to 4.64; *p* < 0.001), lower respiratory tract disorder (aOR 2.99; 95 % CI 1.85 to 4.83; *p* < 0.001) and congenital disorder (aOR 2.73; 95 % CI 1.78 to 4.18; *p* < 0.001). Conversely, French Bulldogs had significantly reduced adjusted odds of 6/32 (18.8 %) grouped-level disorders compared to non-French Bulldogs. These included: complication associated with clinical care (aOR 0.17; 95 % CI 0.09 to 0.32; *p* < 0.001), lethargy (aOR 0.24; 95 % CI 0.11 to 0.51; *p* < 0.001), dental disorder (aOR 0.30; 95 % CI 0.23 to 0.38; *p* < 0.001) and hernia (aOR 0.37; 95 % CI 0.24 to 0.56; *p* < 0.001) (Table 3).

**Table 3:** Grouped-level of diagnostic precision: multivariable logistic regression adjusted odds ratios with corresponding 95 % CIs (confidence intervals) for the most common disorders overall in French Bulldogs and non-French Bulldogs under primary veterinary care at UK practices participating in the VetCompass™ Programme from January 1st 2016 to December 31st, 2016. Model variables accounted for included age, sex-neuter status, at/above or below mean bodyweight, insurance status and veterinary group

Grouped-level disorder	French Bulldog Count (%)	Non-French Bulldog Count (%)	Adjusted odds ratio	95 % CI**	P-value
Upper respiratory tract disorder	304 (10.9)	757 (3.5)	3.80	3.25 to 4.45	**< 0.001**
Spinal cord disorder	30 (1.1)	225 (1.0)	3.00	1.94 to 4.64	**< 0.001**
Lower respiratory tract disorder	27 (1.0)	175 (0.8)	2.99	1.85 to 4.83	**< 0.001**
Congenital disorder	38 (1.4)	61 (0.3)	2.73	1.78 to 4.18	**< 0.001**
Brain disorder	48 (1.7)	374 (1.7)	2.46	1.71 to 3.53	**< 0.001**
Skin disorder	548 (19.7)	2724 (12.5)	2.24	2.00 to 2.50	**< 0.001**
Female reproductive disorder	84 (3.0)	317 (1.5)	1.92	1.47 to 2.50	**< 0.001**
Ear disorder	340 (12.2)	1769 (8.1)	1.86	1.63 to 2.13	**< 0.001**
Collapsed	17 (0.6)	267 (1.2)	1.73	0.95 to 3.16	0.072
Ophthalmological disorder	210 (7.6)	1537 (7.0)	1.47	1.25 to 1.73	**< 0.001**
Urinary system disorder	28 (1.0)	267 (1.2)	1.47	0.96 to 2.25	0.077
Heart disease	44 (1.6)	667 (3.1)	1.43	1.02 to 2.01	**0.041**
Male reproductive disorder	45 (1.6)	198 (0.9)	1.36	0.96 to 1.93	0.082
Anal sac disorder	141 (5.1)	1228 (5.6)	1.31	1.08 to 1.58	**0.006**
Mass	63 (2.3)	1181 (5.4)	1.29	0.98 to 1.71	0.072
Enteropathy	391 (14.1)	2276 (10.4)	1.25	1.10 to 1.41	**0.001**
Musculoskeletal disorder	168 (6.0)	1927 (8.8)	1.18	0.99 to 1.41	0.070
Thin	42 (1.5)	332 (1.5)	1.13	0.79 to 1.61	0.509
Intoxication	25 (0.9)	139 (0.6)	1.13	0.72 to 1.77	0.591
Neoplasia	51 (1.8)	1217 (5.6)	1.04	0.77 to 1.41	0.809
Claw/nail disorder	161 (5.8)	1560 (7.1)	0.94	0.79 to 1.12	0.478
Traumatic injury	93 (3.3)	811 (3.7)	0.81	0.65 to 1.03	0.080
Adverse reaction to drug	19 (0.7)	160 (0.7)	0.77	0.47 to 1.27	0.306
Parasite infestation	105 (3.8)	830 (3.8)	0.76	0.61 to 0.94	**0.012**
Incontinence	4 (0.1)	206 (0.9)	0.76	0.27 to 2.14	0.600
Foreign body	31 (1.1)	279 (1.3)	0.71	0.48 to 1.04	0.080
Endocrine system disorder	2 (0.1)	195 (0.9)	0.56	0.13 to 2.30	0.418
Behavioural issue	65 (2.3)	1164 (5.3)	0.48	0.37 to 0.62	**< 0.001**
Hernia	24 (0.9)	250 (1.1)	0.37	0.24 to 0.56	**< 0.001**
Dental disorder	65 (2.3)	3135 (14.3)	0.30	0.23 to 0.38	**< 0.001**
Lethargy	7 (0.3)	283 (1.3)	0.24	0.11 to 0.51	**< 0.001**
Complication associated with clinical care	10 (0.4)	413 (1.9)	0.17	0.09 to 0.32	**< 0.001**

## Discussion

This study provides an overall evidence base from a primary veterinary care perspective on predispositions and protections to common disorders in French Bulldogs compared to all remaining dogs and identifies some dramatic differences in disorder risks between these two groups of dogs. The demographic results highlight how much younger the French Bulldog population (1.51 years) is in the UK than the remaining dogs (4.48 years) in the wider population and highlights that results from direct (univariable) comparison of disorder risks between French Bulldogs and non-French Bulldogs are likely to be heavily confounded by age [40, 41]. Consequently, risk comparisons between the two breed groups in the current study applied multivariable analytic methods that accounted for age and other confounding variables to increase the reliability of the results. Currently, there are widespread concerns about a reproducibility crisis in veterinary research whereby repeated studies of ostensibly the same research question often reach differing conclusions [42]. The current study aimed to circumvent some of these issues and to achieve more consistent comparison of risk between these two groups of dogs by applying a suite of analyses using a standard approach to the case definitions (i.e. reliance on first opinion veterinary diagnosis) and using multivariable methods to a single but relatively large population of dogs [43]. Comparing relative risks between the breed groups for a series of common disorders based on a single large dataset derived from a random sample of dogs facilitates a more holistic view of health and has highlighted a number of interesting and novel health features of French Bulldogs that are discussed in more detail below.

There is substantial published literature supporting several serious health issues the French Bulldog [13, 14, 19, 44–46]. The UK Kennel Club has such serious concerns for the health of the French Bulldog that the breed is included as a Category 2 on its Breed Watch system, with points of concern for special attention by judges that include respiratory distress, dermatitis in skin folds, prominent eyes, pinched nostrils, incorrect bite and short neck [47]. However, publication bias is a well-recognised phenomenon in science whereby positive findings (e.g. French Bulldogs are predisposed to disease X) are much more likely to be published than the less exciting news of negative findings (e.g. French Bulldogs are *not* predisposed to disease X) [48]. Hence, it is possible that poor health may have been prematurely canonised as ‘fact’ in French Bulldogs and that a fuller exploration of the true overall health of the breed (as carried out in the current study) could lead to different and surprising findings. Nonetheless, based on the prior published view of diminished health in the breed, the current study hypothesised that the count of disorder predispositions in French Bulldogs is greater than the count of disorder protections. The results of the current study show strong support for this position, with French Bulldogs showing 20 predispositions compared with 11 protections from 43 common specific-levels disorders, and French Bulldogs showing 12 predispositions compared with 6 protections from 32 grouped-level disorders. However, the new evidence generated by the current study on a range of protected disorders in French Bulldogs provides some novel nuance to the overall picture of health in the breed and suggests that there are opportunities to move the breed towards a more balanced health profile. For example, one approach would be to redesign the breed by selecting away from conformational extremes that are associated with some of the current predispositions. It is also noteworthy that French Bulldogs differed to non-French Bulldogs in overall propensity (i.e., showed either a predisposition or protection) for 31/43 (72.1 %) of specific-level disorders and 18/31 (56.3 %) of grouped level disorders. Such a high level of disorder risk diversity in either direction between French Bulldogs and all remaining dogs in the wider population emphasises the scale of divergence for the health of the French Bulldog from other dogs and suggests that French Bulldogs should, in many respects, no longer be considered as an ‘average’ or 'typical' dog.

The welfare impacts associated with the many intrinsic health problems of French Bulldogs have been seriously compounded by a dramatic rise in the popularity of the breed, especially over the past decade. During 2020, the French Bulldog recorded their highest puppy registration figures with The Kennel Club since records began, with UK registrations rising by 17 per cent compared to 2019 [1]. Over the last decade, UK registrations for French Bulldogs have risen by 1,682 % [1], There is also evidence from analyses of data from veterinary clinical records of similarly dramatic rising population numbers of French Bulldogs in the wider dog population [44]. The position of the UK Brachycephalic Working Group is that sudden and large increases in population counts can lead to serious welfare issues that may be either predictable or unexpected [49]. Examples of the diversity of such welfare issues for French Bulldogs include worsening breed-related health issues, health deterioration with an ageing population [29], low-welfare breeding (e.g., puppy farms) and sales channels of puppies (e.g., legal and illegal importation [50]), unsuitable ownership profiles, rising rehoming/abandonment, and decreased genetic diversity [51]. The negative impact from these welfare issues that are exacerbated by the current wave of public demand for French bulldogs are considered so great that the UK Brachycephalic Working Group is trying to dissuade potential owners of brachycephalic dogs worldwide with the advice to ‘Stop and think before buying a flat-faced dog’ [5].Following initial domestication around 14,000 years ago [52], dogs were artificially selected towards differing conformations and temperaments to better perform specific roles desired by man such as herding, guarding, hunting or as companion animals [53]. These earlier types of dogs included a wide diversity of conformations matched to their required functions, and those early breeding programmes benefited from recurring outcrossing to improve and prioritise function without much attention being paid to the aesthetic appearance of the dogs [54]. However, rising popularity of dog-showing as a social sport in the late 1800s in Victorian England meant that there was a need to standardise, commoditise and differentiate the pre-existing types or strains of dogs to enable defendable judging decisions at dog shows that were based on appearance, and hence the concept of ‘breed’ in dogs was invented [54]. Written ‘breed standards’ were drafted and debated by breed afficionados to define breeds by their conformations and temperaments [55]. Closed breeding practices were introduced to fix these characteristics within breeds, maintain ‘breed purity’ and promote uniformity [56]. However, over a century later, there is now growing concern and unease that many elements of extreme conformation associated with poorer overall health were unwisely included within some of these breed standards [57–59]. The breed standard for the French Bulldog includes reference to several extreme conformations including ‘skull nearly flat between ears, domed forehead’, ‘lower jaw …. slightly undershot and turned up’, tail … short’, ‘body … cobby’, and ‘the only correct colours are: brindle; fawn and pied’ [60].

Health is challenging to define as a concept, with disorders (i.e., ill-health) being easier to objectively characterise and report [11]. Even in human medicine where persons can self-express their feelings of health, there are multiple reported definitions for health. These include the absence of any disease or impairment, a state that allows the individual to adequately cope with all demands of daily life (implying also the absence of disease and impairment), and a state of balance that an individual has established within himself and between himself and his social and physical environment [61]. However, it is impossible to elicit personal feelings of wellness from dogs and so, the current study accepted that there is currently no single metric that can adequately assess the overall health of a breed. Consequently, the study combined inference based on a series of metrics. One such metric was the proportion of disorders that differed between the two breed groups, followed by a deeper comparison of the counts of predispositions vs. protections [10, 27]. The extent of the differences in odds ratios for these predispositions and protections was also considered. However, it is important to recognise that comparing the relative number of predispositions to protections cannot fully reflect breed health without consideration of the severity and duration of disorders with predispositions and protections [26] and also consideration of disorders that are related to conformation [62].

The propensity (degree) of difference between the overall health of a breed compared with the overall health of all remaining dogs could be used as one indicator of the degree of divergence of individual breeds from the mainstream of current dogs. For breeds where many of these health deviations are related to conformational features, this would provide some evidence that these health deviations are unfortunately associated with extremes of conformation. A previous exploration of predispositions and protections in dogs reported that Labrador Retrievers differed to other dogs in 19/35 (54.3 %) of common disorders, showing predispositions in 12/35 (34.3 %) and protections in 7/35 (20.0 %) [10]. A study using a similar design reported that Staffordshire Bull Terriers differed to other dogs in 9/36 (25.0 %) of common disorders, showing predispositions in 4/36 (11.1 %) and protections in 5/36 (13.9 %) [27]. In support of a view that French Bulldogs have diverged substantially from the mainstream of dogs in the UK and, are in many respects, no longer even a typical dog, is reflected in their higher differces in disorder propensity. Overall, French Bulldogs differed in disorder risk to all remaining dogs for 31/43 (77.1 %) of common disorders. There were 11/43 (25.6 %) specific-level disorders that showed worryingly high high levels of predisposition (ultra-predispositions) with odds over 4 times higher compared with non-French Bulldogs. Several of these ultra-predispositions have previously been linked with aspects of extreme conformation in the breed, including stenotic nares (aOR 42.14) [63, 64], BOAS (aOR 30.89) [65], skin fold dermatitis (aOR 11.18) [66], dystocia (aOR 9.13) [20] and corneal ulceration (aOR 4.38) [59]. Taking a positive view from the association with conformation for this list of ultra-predispositions, it could be argued that awareness of the high contribution of extreme conformation to poor health in French Bulldogs offers substantial potential to reduce the probabilities of these disorders by redesigning the breed away from these extremes of conformation. Selection away from high-risk conformational traits such as skin folds could reap multiple health benefits to the breed, reducing risks of both skin fold dermatitis and corneal ulcers [59, 66], while selection for less extremely brachycephalic muzzle lengths could reduce BOAS and corneal ulcer risk, particularly if combined with selection for wider nostrils for the former [59, 67]. Achieving such conformational changes at a population level for French Bulldogs requires ‘buy-in’ from a wide range of stakeholders including breeders who make the mating selection decisions, and kennel clubs who publish breed standards [60]. However, puppy-buyers also play a key role here, given their potential to alter market dynamics and shift demand towards more moderate conformations. Given that appearance is more influential in the decision to acquire a brachycephalic breed (including French Bulldogs) compared to a non-brachycephalic breed [68], efforts to increase the desirability experienced by prospective puppy-buyers for conformationally moderate French Bulldogs could shift breeders towards producing less extreme conformations in the dogs that they breed.

The current analysis explored differences in probability of diagnosis with at least one disorder during 2016 between French Bulldogs and non-French Bulldogs under primary veterinary care. Multivariable analysis methods were used to account for confounding from other characteristics that might differ between these breed groups such as age, sex-neuter status, at/above or below mean bodyweight, insurance status and veterinary group. Probability of diagnosis with at least one disorder is a relatively new metric to be explored for companion animals and there are several rationales that could potentially explain any differences that are found. In the current study, French Bulldogs showed 0.89 times the adjusted odds of diagnosis with at least one disorder compared with non-French Bulldogs. It is possible that higher odds of diagnosis with at least one disorder in the non-French Bulldogs could reflect poorer health or more complex healthcare needs in these dogs. Alternatively, higher odds of diagnosis may reflect greater recognition of disease by the owners of the non-French Bulldogs such that a higher proportion of veterinary healthcare for these dogs was related to illness rather than to routine prophylactic care.

The current paper highlighted that French Bulldogs were also very different to other breeds in terms of disorders that were protected in the breed i.e., less frequent. There were 11/43 (25.6 %) specific-level disorders where French Bulldogs showed reduced odds, with 4 disorders showing odds ratios below 0.25 that could be categorised as ultra-protections. Much of the current literature on the associations between brachycephalic breeds and health has focused on aspects of reduced health in brachycephalic breeds such as French Bulldogs [11, 69]. However, this approach may not tell the full story and the current paper provides some evidence on aspects of health where French Bulldogs may hold advantage compared to non-French Bulldogs.

There is a growing literature that highlights the depth of the human-animal bond that exists for many owners of French Bulldogs and suggests that the relationships humans share with French Bulldogs are stronger than seen with other breeds of dog [6]. Exploration of these relationships has revealed some of the interpretations and indeed, misinterpretations, of the health and behaviour of brachycephalic breeds [6, 64]. Owners commonly perceive the ‘funny’ personality of these dogs as ‘unique and special’ [6, 7]. In support of this view, undesirable behaviour represented the ultra-protection with the lowest adjusted odds ratio (aOR 0.09) compared with non-French Bulldogs. ‘Undesirable behaviour’ spans a diverse range of behaviours that owners report as unwanted; these behaviours say as much about what owners accept and expect from their dog as they do about the true behavioural patterns of the dog itself [70]. In line with this tendency to show behaviours that are favourable to owners, French Bulldogs were also protected to aggression (aOR 0.64) which may further explain why the breed is considered as an ideal family dog by many owners [7, 68]. Indeed, the description from The Kennel Club of French Bulldogs as ‘the popular clown of the bull breeds’ may find wide appeal with the general public, corroborated by findings of a recent study that explored why owners of brachycephalic breeds, including French Bulldogs, would recommend their breed [7]. Humour and a comical nature were commonly commended behavioural traits (13.1 % of respondents) of brachycephalic breeds, alongside their perceived positive relationships with children (11.8 % of respondents) [7].

There is a large body of evidence to show that the overall syndrome of BOAS as well as its component disorders, including stenotic nares [6, 13, 67, 69, 71], are major issues for the French Bulldog. Despite these reports, it is likely that the true prevalence of BOAS in the French Bulldog population is grossly underestimated by owners and veterinarians. Attribution of clinical signs of BOAS such as snoring and snorting as ‘normal for the breed’ has been documented in brachycephalic dog owners, with 58 % of owners of BOAS-affected dogs failing to recognise that their dog even had a breathing problem [64]. This finding was corroborated by a study of BOAS in French Bulldogs specifically, where 60 % of owners of BOAS-affected dogs were unable to recognize BOAS clinical signs [13]. These findings suggest that many owners of French Bulldogs with breathing problems do not present their dogs to veterinary practices for this problem, and thus BOAS remains an under-recorded disorder. Nevertheless, the current results concur with earlier findings that BOAS is a major issue in French Bulldogs and adds novel data on predisposition to create a fuller picture of the impact of these conditions on the overall health of the French Bulldog. In the current study, French Bulldogs had 42.14 times the adjusted odds of stenotic nares, 30.89 times the adjusted odds of BOAS and 4.88 times the adjusted odds of upper respiratory tract infection compared with non-French Bulldogs. These ultra-predisposition results support current breeding plans that prioritise efforts to reduce the occurrence of BOAS by the use of respiratory function grading schemes [72], breed-specific health schemes [73] and breed health plans [28]. However, it may be that the most effective interventions to reduce the impact of BOAS and its associated disorders will require wider acknowledgement and acceptance by owners and breeders that a more moderate facial conformation with a longer muzzle should become the accepted norm for the breed, given that lower craniofacial ratio (a metric that quantifies relative muzzle length) has been significantly associated with an increased risk of BOAS in two independent populations of French Bulldogs [67, 71].

Corneal ulceration was identified as an ultra-predisposition in the current study, with an adjusted odds ratio of 4.38 in French Bulldogs compared to non-French Bulldogs. Corneal ulceration describes epithelial damage that exposes the corneal stroma [74] and can lead to pain, reflex uveitis, perforation and even loss of the eye [75, 76]. There is substantial corroborating evidence to support strong predisposition to corneal ulceration in the French Bulldog. A previous UK study using primary-care clinical data reported the French Bulldog at 7.25 times the adjusted odds of corneal ulceration compared to crossbred dogs [15] while another UK primary-care study of disorder prevalence reported corneal ulceration as affecting 2.1 % of French Bulldogs annually [44]. French Bulldogs also featured highly in a referral study of corneal ulceration in Japan [77]. Although corneal ulceration can follow a variety of primary (e.g., spontaneous chronic corneal ulceration [78], canine herpes virus-1 [79]) and secondary triggers (e.g., entropion [80], ectopic cilia [81], keratoconjunctivitis sicca [82], corneal degeneration [83], trauma [84] and general anaesthesia [85]), many of these factors are also associated with the brachycephalic phenotype [11, 59, 82, 86]. Common conformational features in French Bulldogs that may promote corneal ulceration include nasal folds (4.84 times the odds of corneal ulcers compared to dogs without nasal folds), a CFR under 0.5 (20.0 times the odds of corneal ulcers compared to CFR > 0.5, with French Bulldog mean CFR 0.18) and relatively wide eyelid apertures [59]. As such, substantial reduction of this ultra-predisposition is likely to require moderation of facial conformation in the wider population of French Bulldogs to protect the corneas of this breed.

Skin fold dermatitis was the fourth highest predisposition of French Bulldogs in the current study, with an adjusted odds ratio of 11.18 compared with non-French Bulldogs. Skin fold dermatitis describes an inflammatory process following abrasion through friction, excessive moisture and reduced ventilation of opposing skin surfaces [87, 88]. Skin apposition in dogs can result from natural features of dogs in general (e.g. axilla) or can result from breed-specific exaggerated conformational features selected in certain breeds such as the skin folds on the face and around the tail base of the French Bulldog [12]. The current ultra-predisposition to skin fold dermatitis in the French Bulldog is supported by earlier studies reporting high occurrence in French Bulldog populations in the UK [44] and Greece [89]. Clinical effects from skin fold dermatitis can vary in severity from mild inflammation with malodour to deep and painful ulceration, and many affected animals are impacted for a large proportion of their lifetime [23, 88]. Applying McGreevy and Nicholas’ [90] considerations for welfare problems in dog breeding, given that facial skin folds run counter to the welfare interests of dogs by risking both dermatological and ophthalmological health, with seemingly no benefit beyond aesthetic appeal, it is difficult to defend the continued promotion of this conformational trait on welfare grounds [90]. Consequently, efforts to encourage selection for, and purchase of, French Bulldogs without skin folds is likely to promote improved welfare for this breed. Such a bold move to prioritise the health needs of the dog over the aesthetic desires of humans would be in line with the vision of the UK Brachycephalic Working Group who state that *‘maximising good health, welfare and temperament overrides all other considerations for dogs’* [5].

The current study had some limitations related to the application of primary-care clinical records as a data resource for epidemiological research that have been reported previously [29, 34]. In addition to these, the current study also applied multiple comparisons between French Bulldogs and non-French Bulldogs throughout the analysis without P-value adjustment that raised the probability of Type I error [91, 92]. However, the current study was more focused on interpreting the overall summative information on disorder predispositions and protections, while the results for each individual disorder should be interpreted in conjunction with the wider previous literature and any novel findings relating to specific disorders in the current study should be treated as results that are hypothesis generating rather than confirmatory [93]. Based on prior evidence that univariable analysis of disorder occurrence in dogs is subject to worryingly high levels of confounding [41], the current study applied an information theory approach to generate standardised multivariable models that aimed to reduce confounding effects [37, 38]. However, it is possible that some residual confounding effects still remained from factors that were unaccounted such as dog-owner bonds, exercise and nutrition [94]. The dogs included in VetCompass studies cover all dogs under primary veterinary care and therefore the current results may not fully reflect the health scores of the specific subset of French Bulldogs that are registered with The Kennel Club.

## Conclusions

This study reports several recognised and novel predispositions and protections in French Bulldogs compared to other dogs in the UK. Based on these differences between the breed groups, the health of French Bulldogs is shown to have diverged substantially from the health of the wider non-French Bulldog dog population and many of these differences appear to be closely associated with common conformational extremes that define the French Bulldog breed. The study provides evidence suggesting that the overall health of French Bulldogs is poorer that the health of non-French Bulldogs but future studies that additionally account for severity and duration for these disorders would add greater nuance to this interpretation. Shifting the typical conformation of French Bulldogs towards a more moderate phenotype is proposed as a logical opportunity to reduce the health issues endemic in the French Bulldog breed.

## Data Availability

The datasets generated during and/or analysed during the current study will be made available at the RVC Research Online repository.

## Abbreviations

aOR: adjusted odds ratio
BHCP: Breed Health and Conservation Plan
BWG: Brachycephalic Working Group
CFR: craniofacial ratio
CI: confidence interval
EPR: electronic patient record
IQR: interquartile range
KC: The Kennel Club
OR: odds ratio
